# Synthesis and Electrospinning of Polycaprolactone from an Aluminium-Based Catalyst: Influence of the Ancillary Ligand and Initiators on Catalytic Efficiency and Fibre Structure

**DOI:** 10.3390/polym11040677

**Published:** 2019-04-13

**Authors:** Ioannis K. Kouparitsas, Elisa Mele, Sara Ronca

**Affiliations:** Department of Materials, Loughborough University, Leicestershire LE11 3TU, UK; i.kouparitsas@lboro.ac.uk (I.K.K.); e.mele2@lboro.ac.uk (E.M.)

**Keywords:** ring-opening polymerisation, functionalised polycaprolactone, electrospinning, porous fibres

## Abstract

In the present study, we investigated the catalytic performance of a 2,2′-methylenebis(6-*tert*-butyl-4-methylphenol) (MDBP)–aluminium complex for the ring-opening polymerisation (ROP) of ε-caprolactone in combination with various alcohols as initiators. Three different alcohols were investigated: 1-adamantanemethanol (**A**), 1H,1H,2H,2H-perfluoro-1-octanol (**F**) and isopropanol (**I**). Samplings of polycaprolactone (PCL) at various reaction times showed a linear increase in the polymer molecular weight with time, with very narrow polydispersity, confirming the living nature of the catalytic system. Scanning electron microscope (SEM) images of electrospun PCL fibre mats produced from 30 wt % dichloromethane/dimethyl sulfoxide solutions showed a high level of surface porosity with a reasonable homogeneity of fibre diameters. The values of the liquid absorption and water contact angle were measured for the electrospun mats, with the **F**-capped PCL consistently showing absorption values up to three times higher than those of PCL samples capped with the other two alcohols, as well as increased hydrophobicity. The nature of the alcohol can influence the surface hydrophobicity and absorption ability of electrospun fibres, demonstrating the possibility of tailoring material properties through controlled polymerisation.

## 1. Introduction

The possibility of fine-tuning the composition and molecular architecture of polymers through synthesis and their precise assembly and organisation through various types of processing is the main reason for their incredibly successful use in such a wide range of applications [1,2].

The field of biomedical sciences has benefitted considerably from this opportunity, as demonstrated by the fast development of novel polymer-based solutions in tissue engineering and drug delivery, with the ability to replicate and/or enhance biological structures and functions [3].

In this respect, the development of biodegradable polymers with fine-tuned composition and their controlled assembly is of substantial interest, whether the material needs to offer either mechanical support in a timeframe that is compatible with the restoration of injured tissue [4], or the controlled release of compounds [5]. Polycaprolactone (PCL) has been the preferred choice for many of these applications thanks to its biocompatibility, favourable mechanical properties and degradation kinetics that can be tailored by varying the molecular weight and/or crystallinity of the polymer [6].

**Figure 1:**
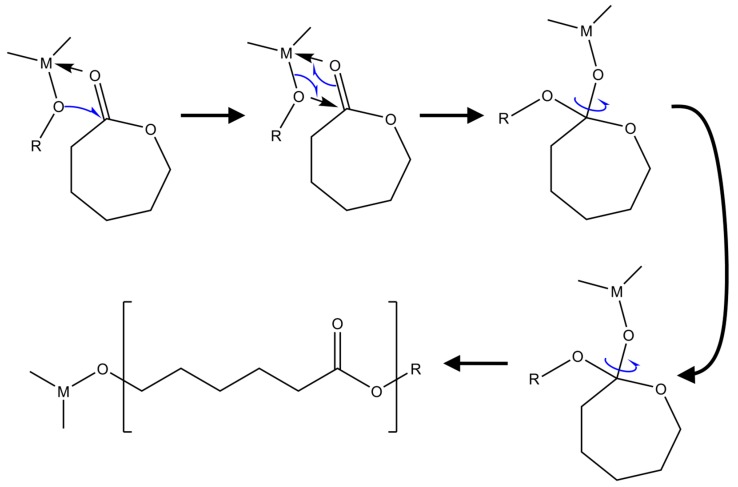
Ring-opening polymerisation mechanism of ε-caprolactone mediated by a metal catalyst.

PCL is usually synthesised by the ring-opening polymerisation (ROP) of ε-caprolactone (ε-CL) [7], a process that can be mediated by a metal catalyst, according to the mechanism illustrated in Figure 1.

Tin octoate is certainly the most common catalyst for the ROP of ε-CL thanks to its commercial availability, easier handling and solubility in most commonly used organic solvents. An alcohol is required to initiate the reaction. The main issue when using tin octoate is that it necessitates high temperatures, which may favour intermolecular and intramolecular esterification, broadening the polydispersity of the resulting polymer. Moreover, for biomedical applications, the use of tin may be of concern due to the cytotoxicity of this metal in some forms [8,9].

A possible alternative catalyst was described by Hamitou et al. [10]. The authors elucidated the reaction mechanism of aluminium-based catalysts activated by alcohols in the polymerisation of lactones, showing that these species can produce almost mono-dispersed polymers at room temperature and with high conversion. Interestingly, the reaction mechanism implies that the resulting polyester will always have one hydroxyl end group resulting from the hydrolysis of the catalytic mixture, while the nature of the other end group will be fixed by the initial structure of the catalyst alkoxide groups, i.e., by the choice of the alcohol/initiator. For these reasons, and due to the inherent low toxicity of aluminium oxide compounds [11], we decided to focus our attention on this class of catalysts. We have reported previously [12] on the possibility to synthesise PCL with photo-curable vinyl groups using an aluminium catalyst with 2,6-di-*tert*-butyl-4-methylphenol (BHT), similar to that reported by Akatsuka et al. in 1995 [13].

For biomedical applications, electrospinning is widely used to process PCL in the form of micro- or nano-fibres, showing potential as scaffolds for tissue engineering. The high surface area of electrospun fibres is advantageous in increasing load capacity [14], even more if the fibres exhibit a porous surface instead of a smooth one.

The possibility to electrospin PCL into porous fibres holds considerable interest for such applications, as surface porosity or internal channels can promote cell adhesion and the transport of nutrients in tissue-engineering applications [15]. Most electrospinning approaches to achieve fibre porosity involve the use of systems composed of polymers or solvent blends [16], sometimes in the presence of specific environmental conditions [17], such as increased humidity [18], or with the help of additional post-processing [19].

Recently, Katsogiannis et al. reported on the possibility to produce porous electrospun PCL fibres by the careful selection of electrospinning parameters and solvent composition [20]. The authors showed that electrospinning 15 *w*/*v* % solutions of PCL in mixtures of chloroform and dimethyl sulfoxide could directly originate porous, bead-free fibres, but at the expense of fibre homogeneity and dimensions.

Huang et al. reported on the possibility of generating either surface or internal porosity when electrospinning systems based on polylactic acid (PLA) in concentrations of 15 *w*/*v* % in various solvent combinations that included ethanol, dimethyl sulfoxide and chloroform [17]. The authors showed that porosity played a more important role in oil sorption than fibre diameter.

In the present paper, we report a combined study on the synthesis and processing of polycaprolactone to elucidate: (i) the influence of the ancillary ligand structure and nature of the initiator on the catalytic performance of the chosen aluminium catalyst, and (ii) the effect that the functional groups introduced via the initiators have on the structure, hydrophobicity and liquid-loading ability of resulting electrospun fibres.

## 2. Materials and Methods

All reagents, except for anhydrous toluene (purchased from Sigma-Aldrich, Gillingham, UK), were supplied by Fisher Scientific (Loughborough, UK), Acros Organics (Loughborough, UK) and used as received unless otherwise stated. All glassware used for the preparation of catalysts and for polymer synthesis was dried overnight in an oven at 125 °C and purged with 3 vacuum-nitrogen cycles before use.

**Synthesis:** The catalyst preparation and polymerisation reactions were performed under a controlled atmosphere (either nitrogen or argon). Catalyst preparation was conducted in a glove box under argon atmosphere, by reacting equimolar amounts of tri-isobutyl aluminium (TibAl, solution in toluene 1.1 M) and 2,2′-methylenebis(6-*tert*-butyl-4-methylphenol) (MDBP) dissolved in anhydrous toluene under stirring for 10 min at room temperature in a Schlenk tube. Subsequently the desired alcohol (1-adamantanemethanol, 1H,1H,2H,2H-perfluoro-1-octanol or isopropanol, indicated respectively as the **A**, **F** and **I** initiator) was added in an equimolar amount to the Al-MDBP solution, under an additional 10 min of stirring. After that time, the solution was injected into a 0.5 L jacketed Pyrex reactor containing a solution of ε-caprolactone (typically 20 g, dried over molecular sieves, 3 Å) in anhydrous toluene (250 mL) at 40 °C under a nitrogen atmosphere and the reaction was kept under magnetic stirring for up to 5 h. The reaction was quenched by slowly pouring the contents of the reactor into a beaker containing 500 mL of cold methanol. The resulting polymers were filtered and dried overnight in a vacuum oven at 30 °C. Samplings (~10 mL solution per sampling) for the kinetic study were taken using a syringe with needles that had been oven-dried and purged with nitrogen beforehand. Samplings were immediately quenched in an excess of methanol and the precipitated polymers were dried and analysed as described below.

**Electrospinning:** Dichloromethane (DCM) and dimethyl sulfoxide (DMSO) were used as solvents in a ratio of 6:1 *v*/*v* to prepare the electrospinning PCL solutions at 10, 20 and 30 wt %. The electrospinning parameters used were: flow rate of 4.5 mL/h, voltage of 8–10 kV and tip-collector distance of 15 cm. All mats obtained were coherent and mechanically stable enough to be easily detached from the aluminium collector and cut in desired shapes/sizes for further testing (contact angle, liquid absorption).

**Characterisation**: Differential scanning calorimetry (DSC), gel permeation chromatography (GPC), scanning electron microscopy (SEM), liquid absorption and water contact angle (WCA) studies were employed to characterise the materials used.

DSC: The thermal properties of the polymer powders were analysed using a TA Instruments Q2000 DSC (TA instruments, Elstree, UK). The samples were weighed and placed in Tzero aluminium pans with Tzero lids. Between 4 and 6 mg of polymer were weighted on a high-precision balance (±0.001 mg) and analysed from −90 °C to 130 °C with a heating/cooling/heating ramp at a rate of 10 °C/min. From the resulting data the peak produced at the first melting temperature (*T*_m1_) and enthalpy of the first heating cycle (Δ*H*_m1_ in J/g) as well as the peak crystallisation temperature (*T*_c_) of the cooling cycle were recorded. Using the enthalpy of fusion for the samples along with the enthalpy of fusion for 100% crystalline PCL, 135.6 J/g [21], the % crystallinity of each sample was calculated using Equation (1):

*X*_c_ % = (sample enthalpy of fusion/100% crystalline PCL enthalpy of fusion) × 100 (1)

GPC: Measurements were conducted on an Agilent 1260 Infinity (Agilent, Cheshire, UK) equipped with a refraction index (RI) detector and calibrated with GPC PS standard provided by Agilent. Solutions of 1 mg/mL of polymer in chloroform with added 3% *v*/*v* TEA (triethylamine) were prepared. Analysis of the results was conducted using Mark-Houwink constants for PCL in chloroform at 30 °C, *K* = 12.98 and α = 0.828 [22].

SEM: In order to assess the porosity of the fibres, the samples were observed using a Hitachi TM3030 benchtop SEM (Hitachi, London, UK). Pieces of the fibre mats were coated using a Quorum Q150R S with Au/Pd (90 s, 80:20) before imaging.

Liquid absorption: For the absorption tests, olive oil, manuka essential oil, black pepper oil, dodecane and silicone oil were employed. A portion of electrospun mat was cut with scissors, weighed on a precision balance, immersed in the liquids and then weighed again after immersion. The absorption ability was reported as the weight of absorbed liquid/initial weight of the mat.

WCA: Contact angle measurements were conducted using a Dataphysics OCA 20 contact angle machine (Dataphysics, Filderstadt, Germany). A drop of 2 μL of distilled water was placed onto flat pieces of mats using the sessile drop method. The resulting contact angle was measured via the accompanied camera/software. An average was calculated from five measurements for each sample.

## 3. Results and Discussion

We have previously reported on the catalytic performance of an Al-(BHT)_2_ catalyst in the presence of suitable alcohol initiators [12].

**Figure 2:**
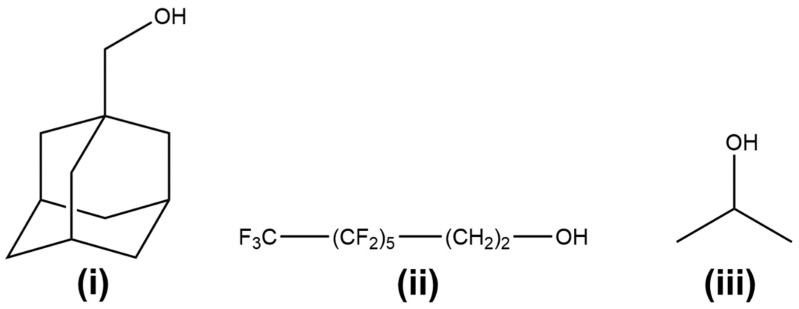
Alcohol initiators utilised for the activation of tri-isobutyl aluminium: 1-adamantanemethanol (**i**), 1H,1H,2H,2H-perfluoro-1-octanol (**ii**) and isopropanol (**iii**).

In the present study, we wanted to assess the steric/electronic influence of the initiator on the catalyst performance and resulting polymer properties, especially in terms of hydrophobicity and liquid absorption ability when processed in the form of electrospun fibres. For this purpose, three different initiators were selected: 1-adamantanemethanol (**A**), 1H,1H,2H,2H-perfluoro-1-octanol (**F**) and isopropanol (**I**), shown in Figure 2. Isopropanol is a common alcohol used as an initiator with Al catalytic systems [7]; perfluoro-octanol was chosen to potentially enhance the hydrophobicity of the PCL chain [23,24], while adamantane-terminated PCL has been recently shown to produce non-covalently bound block-copolymers with cyclodextrin-polyvinylpyrrolidone (CD-PVP) [25].

Preliminary attempts at using the Al-(BHT)_2_ catalyst in combination with **F** were not successful, possibly due to the high steric hindrance of the two BHT groups and the alcohol itself; moreover, aluminium derivatives of monodentate bulky phenols have been reported to show a decrease in catalytic activity in the presence of excess alcohol (isopropanol specifically), due to the metathesis occurring between the aluminium phenolate and isopropanol [26]. A more stable version of an Al-phenolate catalyst was reported by Ko et al. [27]. The authors tested the polymerisation performance of an organo-aluminium in combination with a bidentate ligand such as 2,2-ethylidenebis(4,6-di-*tert*-butylphenol), demonstrating that this catalytic system was able to keep a living character (i.e., one catalytic site producing one polymeric chain) in the polymerisation of caprolactone for up to 32 h.

We decided to test the catalytic efficiency of a system similar to the one described by Ko, with the use of 2,2′-methylenebis(6-*tert*-butyl-4-methylphenol) (MDBP) as the ancillary ligand for the aluminium. The other possible advantage of using a bidentate ligand instead of two monodentate ones is the increased accessibility of the metal’s frontal space (a well-established concept in the design of metallocene catalysts for olefin polymerisation [28]), thus allowing an easier reaction with sterically hindered alcohols such as **F** and **A**.

In Table 1, the results of a test reaction performed with the catalytic system Al-MDBP activated with isopropanol (Al-MDBP-**I**) are reported. Samplings of the catalytic solution (~10 mL) were taken at various times up to 210 min, quenched in cold methanol, and the resulting polymers were characterised via GPC to follow the progressive increase in molecular weight (number average, *M*_n_, and weight average, *M*_w_) and confirm the living character of the system.

**Figure 3:**
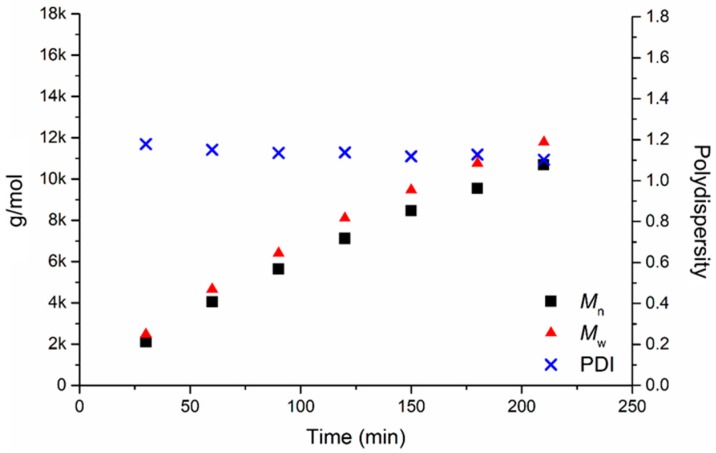
Values of *M*_n_, *M*_w_ and PDI as a function of time in the polymerisation of ε-CL with the Al-MDBP-**I** system.

As evident from Figure 3, the molecular weight of PCL increased in a linear trend with time, with very narrow polydispersity indexes (PDI) extending between 1.10 and 1.18, thus confirming the living nature of the catalytic system.

**Figure 4:**
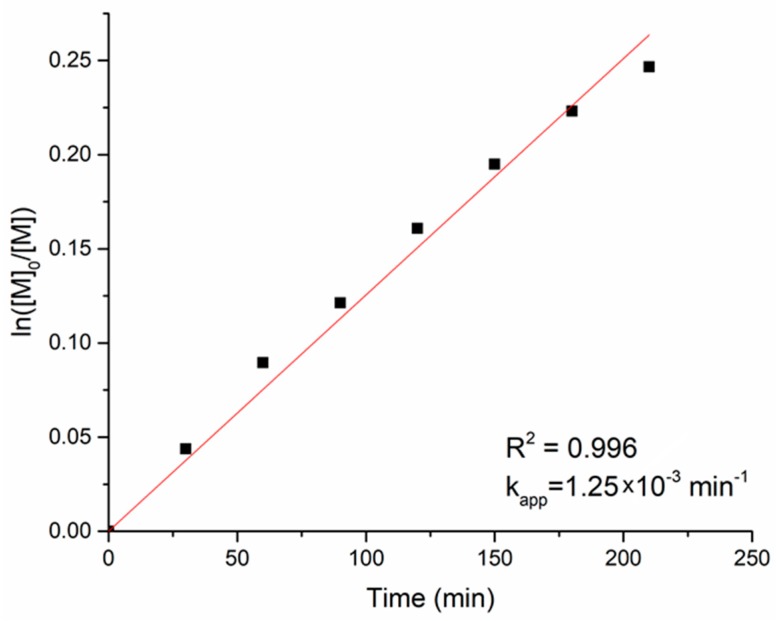
Kinetics of the ε-CL polymerisation in toluene at 40 °C, initiated by Al-MDBP-**I**.

The graph in Figure 4 shows the variation of ln([M]_0_/[M]) with time, where [M]_0_ is the monomer concentration at the beginning of the reaction and [M] is the concentration at any given time *t*. The polymerisation rate showed a linear dependence of ln([M]_0_/[M]) versus time, according to Equation (2) [29]:

ln([M]_0_/[M]) = *k*_app_ × *t* (2)

The linearity confirmed that the reaction was first order with respect to the monomer, with a kinetic rate constant *k*_app_ = 1.25 × 10^−3^ min^−1^. This value is close to those observed in similar systems by Dubois et al. [29].

In Table 2, the reaction conditions used for the synthesis of three PCL samples in the presence of the chosen initiators **A**, **F** and **I** and the resulting values of molecular weights and distributions as obtained from GPC measurements are reported.

Up to 20 g of PCL were synthesised in 5 h, with a 0.5 L reactor; we foresee that the reaction could be scaled up to larger amounts by adjusting the reactor volume and corresponding reagents.

It is interesting to note that for PCL_A and PCL_I, the moles of the active sites calculated from the ratio Y/*M*_w_, where Y is polymer yield (in g) obtained after quenching, and *M*_w_ is the average molecular weight (in g/mol), agreed rather closely with the moles of catalyst introduced into the system. This confirms that all aluminium sites were correctly activated by the initiators and only one chain was produced for each active site. In the case of PCL_F, the active sites were about half of the aluminium sites introduced, however we attributed this result to the lower solubility of **F** in toluene.

Another interesting observation is that the molecular weight of PCL_I was slightly higher than that of PCL_F and PCL_A, suggesting either a faster initiation at the less sterically-hindered active sites, faster kinetics, or a combination of both. Studies of the kinetics of polymerisation as a function of the initiator are underway to confirm the most likely reason, and to show how it is possible to target higher or lower molecular weights by a careful choice of CL/initiator and reaction times.

In Table 3, the melting/crystallisation temperatures and nascent crystallinities for the three analysed samples are reported. All samples show melting in the typical range of ~60 °C, with high crystallinities, reaching over 77% for PCL_F. These values agree with previously observed values for PCL samples of similar molecular weight [30].

**Figure 5:**
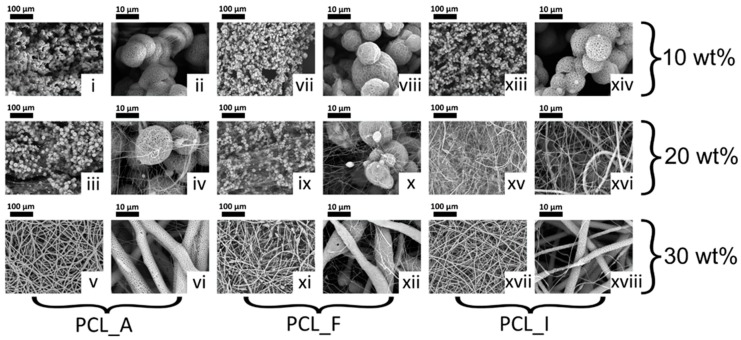
Scanning electron microscope (SEM) micrographs of electrospun samples of PCL_A (**i**–**vi**), PCL_F (**vii**–**xii**) and PCL_I (**xiii**–**xviii**) at 10, 20 and 30 wt % solutions in DCM/DMSO (6:1 *v*/*v*). Magnifications at 500× (100 μm bar) and 5000× (10 μm bar).

The three PCL samples were electrospun from solutions of DCM/DMSO (6:1 *v*/*v*) at concentrations ranging from 10 wt % to 30 wt %, and the SEM images of the resulting fibres are shown in Figure 5. PCL_A and PCL_F only demonstrated bead formation at the lowest concentration, while some proto-fibres were identified at 20 wt %. Well-formed fibres could only be obtained at the highest concentration of 30 wt %. For PCL_I, fibre formation started at the intermediate concentration, possibly due to the slightly higher molecular weight of the polymer.

**Figure 6:**
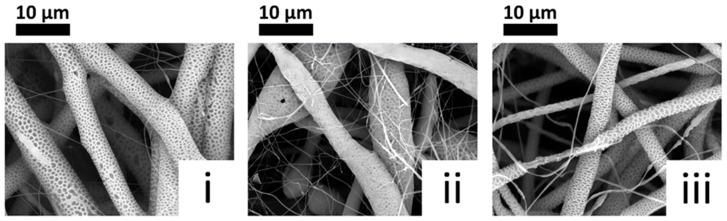
SEM micrographs at 5000× magnification of PCL_A (**i**), PCL_F (**ii**) and PCL_I (**iii**) electrospun from 30 wt % solutions in DCM/DMSO (6:1 *v*/*v*).

A close-up of the fibres obtained at 30 wt % is shown in Figure 6. It is interesting to note that the fibres showed a high level of surface porosity, while maintaining a reasonable size homogeneity. Fibre diameters were in the range of tenths of microns, with pores in the range of microns.

Further to the discussion in the introduction, the presence of porosity in electrospun fibres can be associated with the fast evaporation of the solvent during electrospinning [31]. In our case, the process may have been more pronounced due to the use of more concentrated solutions, thus reducing the amount of solvent and consequently the time needed for it to evaporate.

The high surface area resulting from the presence of pores, associated with the inherent hydrophobicity of PCL, make these electrospun mats ideal for the absorption of oils, a characteristic that has been widely explored in the case of biomedical applications when antimicrobial essential oils are used [32,33].

We therefore tested the ability of the electrospun samples to absorb a variety of hydrophobic liquids and calculated their absorption ability *Q* according to Equation (3):

*Q* = (*W* − *W*_0_)/*W*_0_ (3)

where *W*_0_ is the initial mat weight and *W* the mat weight after absorption.

**Figure 7:**
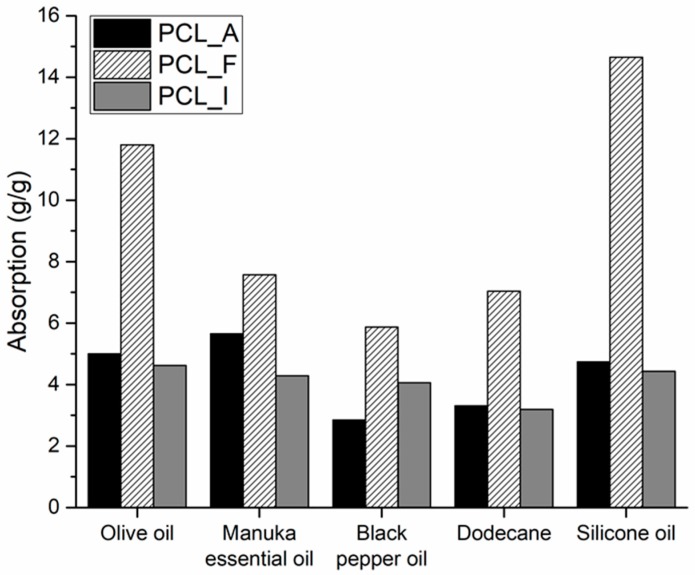
Absorption of hydrophobic liquids, *Q* (g/g), for PCL_A, PCL_F and PCL_I.

Figure 7 shows the absorption values for the fibre mats produced from the three PCL samples, with respect to five different hydrophobic liquids, namely olive oil, manuka essential oil, black pepper oil, dodecane and silicone oil. The values of *Q* range between 3 and 15 g/g, with PCL_F consistently showing absorption values that were double or even triple that of PCL_A and PCL_I.

Reshmi et al. have reported similar values of absorption for PCL fibres, both pure or with added beeswax to improve the efficiency of oil separation [34]. The authors mention the role of beeswax in increasing surface roughness and hydrophobicity, resulting in higher absorption capacities of the PCL/beeswax electrospun blends when compared to pure PCL.

In our case, the advantage was the possibility to enhance the hydrophobicity of the material with the direct introduction of a hydrophobic group into the polymeric chain itself, thus removing the need for additional fillers or components.

**Figure 8:**
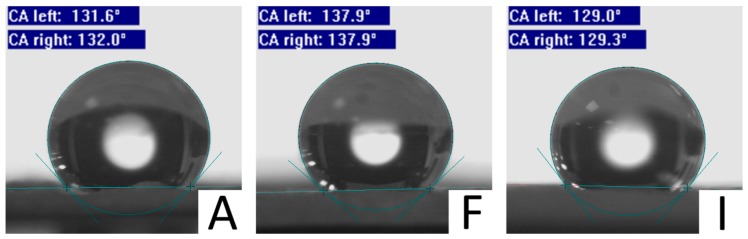
Images of water contact angle (CA) measurements for each sample. Average values from five measurements are presented in Table 4.

The enhanced hydrophobic character of PCL_F mats was confirmed by water contact angle measurements, as shown in Table 4 and Figure 8.

## 4. Conclusions

In this paper, we reported on the synthesis of polycaprolactone (PCL) with an aluminium-based catalyst in combination with various alcohols as initiators, followed by the electrospinning of the resulting polymers. The catalytic system showed a living character under the experimental conditions used (toluene solution, 40 °C, 5 h reaction), allowing for the controlled synthesis of almost mono-modal PCL with defined chain-end groups that originated from the chosen initiator. Larger, more sterically-hindered initiators, such as 1-adamantanemethanol and 1H,1H,2H,2H-perfluoro-1-octanol result in lower molecular weight polymers for the same reaction time when compared to smaller, less sterically hindered initiators such as isopropanol, suggesting an effect of the initiator on the induction time and/or the polymerisation rate.

All PCL samples could be electrospun in homogeneous, micro-sized fibres from rather concentrated (30 wt %) DCM/DSMO solutions. All fibres showed a high level of surface porosity that has been exploited to assess their ability to absorb hydrophobic liquids. The electrospun mats produced from the perfluoro-octanol-substituted PCL showed the highest hydrophobicity from water contact angle measurements, as well as the highest liquid absorption ability.

The results suggest that the chosen catalytic system could be efficiently used to produce PCL with controlled molecular weights and with the desired functionalities successfully introduced without the need for additional post-processing.

## Figures and Tables

**Table 1:** *M*_n_, *M*_w_ and polydispersity indices (PDI) of samplings taken at various times for the test polymerisation of ε-CL with the catalytic system Al-2,2′-methylenebis(6-*tert*-butyl-4-methylphenol)- isopropanol (Al-MDBP-**I**) in toluene ^a^.

Time (min)	*M*_n_ (g mol^−1^)	*M*_w_ (g mol^−1^)	PDI	ε-CL Conversion (%)	Theoretical *M*_w_ (g mol^−1^)
30	2106	2481	1.178	4	2200
60	4051	4662	1.151	8	4300
90	5646	6411	1.135	11	6000
120	7120	8101	1.138	15	8100
150	8459	9467	1.119	18	9700
180	9547	10,765	1.128	22	11,900
210	10,688	11,782	1.102	25	13,500

^a^ Reaction conditions: temperature = 40 °C; solvent = 250 mL toluene; Al-MDBP: 0.37 mmol; **I**: 0.37 mmol; ε-CL: 0.176 mol; ratio CL:Al:I = 480:1:1.

**Table 2:** Reaction conditions and resulting molecular weights/distributions listed by initiator type ^a^.

Sample	Al-MDBP-In ^b^ (mmol)	CL (mol)	CL:Al	Conv. (%)	Yield (g)	*M*_n_ (kg/mol)	*M*_w_ (kg/mol)	Al* ^c^ (mmol)	PDI
PCL_A	0.37	0.355	960:1	23	9	17	20	~0.4	1.14
PCL_F	0.74	0.176	240:1	40	8	18	21	~0.4	1.12
PCL_I	0.74	0.176	240:1	~100	20	22	27	~0.7	1.19

^a^ All reactions performed in 250 mL of toluene, at 40 °C, for 5 h; ^b^ In = Initiator: PCL_A: 1-adamantanemethanol, PCL_F: perfluoro-octanol; PCL_I: isopropanol; ^c^ Al*: calculated active sites.

**Table 3:** Differential scanning calorimetry (DSC) results showing melting/crystallisation temperatures and melting enthalpies. Nascent crystallinity was calculated from the melting enthalpy using ∆*H*^°^_m_ = 135.6 J/g [21].

Sample	*T*_m1_ (°C)	*T*_c_ (°C)	Δ*H*_m1_ (J/g)	*X*_c_ %
PCL_A	57.2	36.4	92	67.8
PCL_F	62.7	35.7	105.2	77.6
PCL_I	61.9	34.1	102.7	75.7

**Table 4:** Water contact angle for PCL mats electrospun from 30 wt % solutions.

Sample	Water Contact Angle (°)
PCL_A	131.9 ± 1.0
PCL_F	137.6 ± 1.3
PCL_I	129.6 ± 3.7
